# Investigating the Association Between rs2439302 Polymorphism and Thyroid Cancer: A Systematic Review and Meta-Analysis

**DOI:** 10.3389/fsurg.2022.877206

**Published:** 2022-04-26

**Authors:** Yawen Guo, Wanchen Zhang, Ru He, Chuanming Zheng, Xuefeng Liu, Minghua Ge, Jiajie Xu

**Affiliations:** ^1^Department of Head and Neck Surgery, Otolaryngology & Head and Neck Center, Cancer Center, Zhejiang Provincial People's Hospital (Affiliated People's Hospital, Hangzhou Medical College), Hangzhou, China; ^2^Department of Public Health, Zhejiang University School of Medicine, Hangzhou, China; ^3^Key Laboratory of Endocrine Gland Diseases of Zhejiang Province, Hangzhou, China; ^4^Second Clinical Medical College, Zhejiang Chinese Medical University, Hangzhou, China; ^5^School of Basic Medical Sciences and Forensic Medicine, Hangzhou Medical College, Hangzhou, China; ^6^Neck and Breast Department 3, Tumour Hospital of Mudanjiang City, Mudanjiang, China

**Keywords:** thyroid cancer, rs2439302, meta-analysis, single nucleotide polymorphism, genome-wide association studies

## Abstract

**Background and Aims:**

The extent of surgical treatment for most patients with thyroid cancer (TC) remains controversial and varies widely. As an emerging technology, genetic testing facilitates tumor typing and disease progression monitoring and is expected to influence the choice of surgical approach for patients with TC. Recent genome-wide association studies (GWASs) have identified that rs2439302 (8p12) variants near NRG1 are associated with TC risk; however, the results remain inconclusive. Therefore, we aimed to perform a meta-analysis to clarify the association between rs2439302 variants and the risk of TC.

**Methods:**

We search eligible studies using Pubmed, Scopus, Embase, Web of Science, and Cochrane library by July 2021. We analyzed the pooled OR and the corresponding 95% confidence interval (95% CI) of the included studies and then conducted subgroup analysis according to the ethnicity. We also performed a sensitivity analysis to validate the findings.

**Results:**

This meta-analysis finally included 7 studies involving 6,090 cases and 14,461 controls. Results showed that the G allele of the rs2439302 polymorphism was a significant risk factor of TC in Allele (G/C), Dominant (GG+GC/CC), Recessive (GG/GC+CC), Homozygote (GG/CC), Heterozygote (GC/CC) models, with pooled ORs of 1.38 (95%CI, 1.31–1.45), 1.51 (95%CI, 1.41–1.62), 1.52 (95%CI, 1.40–1.66), 1.90 (95%CI, 1.71–2.10), and 1.40 (95%CI, 1.30–1.51), respectively. The subgroup analysis showed that rs2439302 polymorphism was associated with higher TC risk in different ethnicities with OR > 1. The sensitivity analysis exhibited that the results were stable by omitting any included studies.

**Conclusions:**

The study revealed that rs2439302 variants were associated with higher TC risk and may have a major influence on the choice of operative approach for patients with TC.

## Introduction

Being the most prevalent malignancy in the endocrine system, thyroid cancer (TC) has become a serious disease threatening the health of the human being. Nearly 52,890 cases of TC were predicted to be diagnosed in the United States in 2020 (1). Moreover, the incidence of TC was among the top ten of the malignant tumor spectrums in China, accounting for 7.7% and 5.12% of the total cases in 2018 and 2015, respectively (2–4). The first line treatment for TC is surgery, except for certain cases of anaplastic TC (ATC). However, the extent of thyroidectomy and lymph node dissection especially for papillary TC remains controversial and varied (5). In the emerging era of genomic and precision medicine, genomic analysis relies on the patient's tumor tissue as a component of the diagnosis and treatment (6, 7), but our understanding of this genetic characteristic of TC is limited.

With the sharing of the single nucleotide polymorphism (SNPs) database represented by the International HapMap Project and the establishment and improvement of high-throughput genotyping technology, Genome-Wide Association Studies (GWASs) have become an important strategy for studying genetic mechanisms of complex diseases such as TC (8–10). Some TC risk alleles, such as 2q35 (rs966423), 9q22 (rs965513), 8p12 (rs2439302), 8q24 (rs6983267), and 14q13 (rs944289 and rs116909374), have been found based on several GWASs and candidate studies on Europeans (8, 9, 11, 12). Multiple studies of these variants in the British, United States, Japanese, and Chinese populations confirmed the association between these variants and the TC risk (11, 13, 14).

The SNP rs2439302 was within the first intron of NRG1 (gene encoding neuregulin 1) on 8p12. Julius Gudmundsson first demonstrated that rs2439302 was significantly correlated with TC (OR = 1.36; P combined = 2.0 × 10^−9^) in 2011. Subsequently, associations between the rs2439302 polymorphism with TC risk and clinical parameters in different populations have been investigated (11, 14–22); however, the published results are inconsistent. Positive associations between rs2439302 polymorphisms and TC were found in Asian (14, 16, 17, 22) and Caucasian (8, 11, 18) populations. However, one study reported a marginal association between rs2439302 and TC in Columbia's population. To our knowledge, this article is the first meta-analysis carried out to clarify whether rs2439302 variants are correlated with TC risk.

## Materials and Methods

### Data Source and Keyword Selection

According to the Preferred Reporting Items for Systematic Reviews and Meta-Analyses (PRISMA), we conducted the systematic literature search for relevant articles from databases including Pubmed, Scopus, Embase, Web of Science, and Cochrane library, by the end of July 2021. Since the study only extracted data from published studies, ethical approval was not required. The review was not registered.

The search terms we used in this study were as follows: “rs2439302,” “8p12,” “polymorphism,” “variation,” “variant,” “thyroid cancer,” “carcinoma of thyroid,” “thyroid carcinoma,” “thyroid neoplasm,” and “thyroid malignancy.” All the search records were limited to human studies and the language was restricted to English. The inclusion criteria were as follows: (1) used a case-control design; (2) evaluated the association between rs2439302 polymorphism and TC; (3) provided the number of rs2439302 genotypes or provided sufficient data to calculate the number of rs2439302 genotypes; (4) provided the odds ratios (OR) estimates and their 95% CIs or provided sufficient data to calculate the OR and 95% CI; (5) published in English or Chinese. We excluded the following studies: (1) duplicated publications; (2) irrelevant studies; (3) meta-analysis or review; (4) no access for full text; (5) case reports; (6) no associated data for extraction. As for the articles in which no relevant data are available, we contacted the corresponding authors to achieve the original data.

### Data Extraction

An independent review of the included studies by 2 scientists was performed. The following parameters were extracted from the studies: the family name of the first author, the year of publication, the country of the population, ethnicity of the population, detailed number of the population, and specific genotype frequency of the population.

### Statistical Analysis

We used the odds ratios (OR) and 95% confidence interval (95% CI) for the assessment of the association between the rs2439302 variants and TC. Five different models used were as follows: (1) G vs. C (allele model), (2) GG plus CG vs. CC (dominant model), (3) GG vs. CC plus CG (recessive model), (4) GG vs. CC (homozygous model), and (5) GC vs. CC (heterozygous model). The Chi-square test was used to analyze the Hardy-Weinberg equilibrium (HWE) for the control. Chi-square-based Q statistic and *I*^2^ test were used for assessing the heterogeneity between studies. Higher *I*^2^ values indicated higher levels of heterogeneity (low, moderate, large, and extreme heterogeneity corresponded to 0–25%, 25–50%, 50–75%, and 75–100%, respectively). The fixed-effects model was used when the *p*-value was >0.05, while the random-effects model was used when the *p*-value was <0.05. The Egger's test and Begg's funnel plot were used to analyze the publication bias. A sensitivity assessment was performed to reveal whether the ethnicity exerted an effect on the findings. All *p*-values were two-sided, *p* < 0.05 were considered statistically significant. The statistical analysis was conducted using R software.

## Results

### Study Characteristics

**Figure 1:**
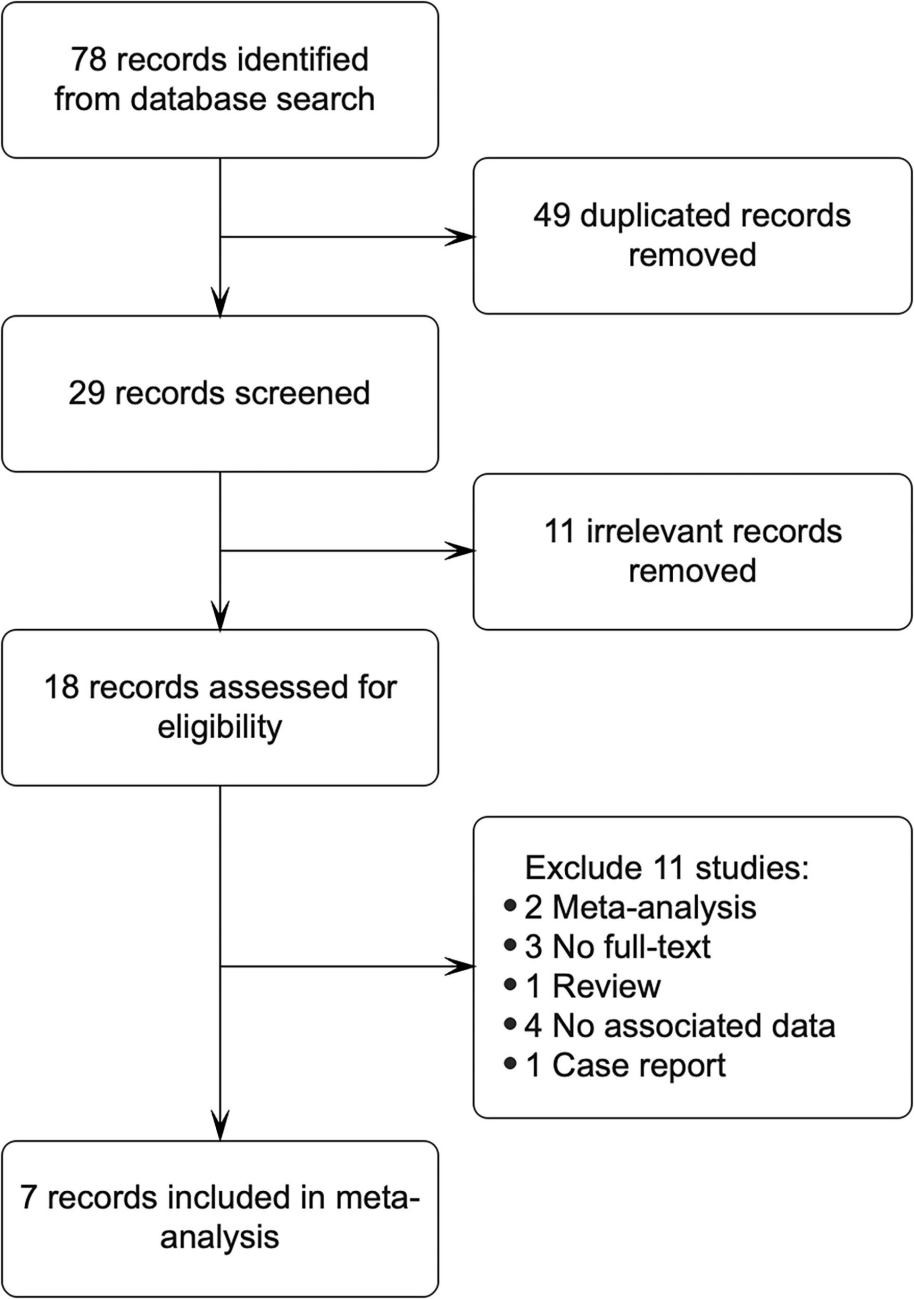
The flow gram of the study was shown.

This meta-analysis included 78 articles from Pubmed, Scopus, Embase, Web of Science, and Cochrane library, obtained by using different combinations of key terms. Overall, 49 records were excluded as they were duplicates; 11 records were irrelevant excluded after reviewing titles and abstracts; 11 records were removed based on the following defect: meta-analysis (*n* = 2), no full text (*n* = 3), review (*n* = 1), no associated data (*n* = 4), and case report (*n* = 1). Finally, 7 studies involving 6,090 cases and 14,461 controls met our inclusion criteria (8, 11, 14, 16–18, 23), and 1 of them has insufficient data, the original data were obtained by contacting the corresponding authors (17). All studies had case-control study designs. We made a flow diagram to show the detailed process of the study (Figure 1).

The characteristics of the eligible studies are shown in Table 1. Among all these 7 studies, 4 studies were from Asia, and 3 studies were from Western countries. Moreover, in all these 5 studies, the genotype distribution in the controls was consistent with the Hardy–Weinberg equilibrium.

**Table 1:** Characteristics of included studies in the meta-analysis.

**Author**	**Year**	**Group**	**Country**	**Thyroid cancer**			**Control**			**HWE**	**Ethnicity**
				**GG**	**CG**	**CC**	**GG**	**CG**	**CC**		
Gudmundsson	2011	Case control	Mixed	317	563	254	1,051	2,734	1,840	Y	Other
Liyanarachchi	2013	Case control	Mixed	584	978	410	565	1,226	666	Y	Other
Wang	2013	Case control	China	49	295	501	34	289	682	Y	Asian
Wei	2015	Case control	China	49	291	498	15	143	343	Y	Asian
Rogounovitch	2015	Case control	Japan	31	196	308	104	855	1,765	Y	Asian
Estrada-Florez	2016	Case control	USA	74	152	55	285	550	306	Y	Other
Mussazhanova	2021	Case control	Japan	90	238	157	110	446	452	Y	Asian

### Correlation Between rs2439302 Polymorphism and TC Risk

**Figure 2:**
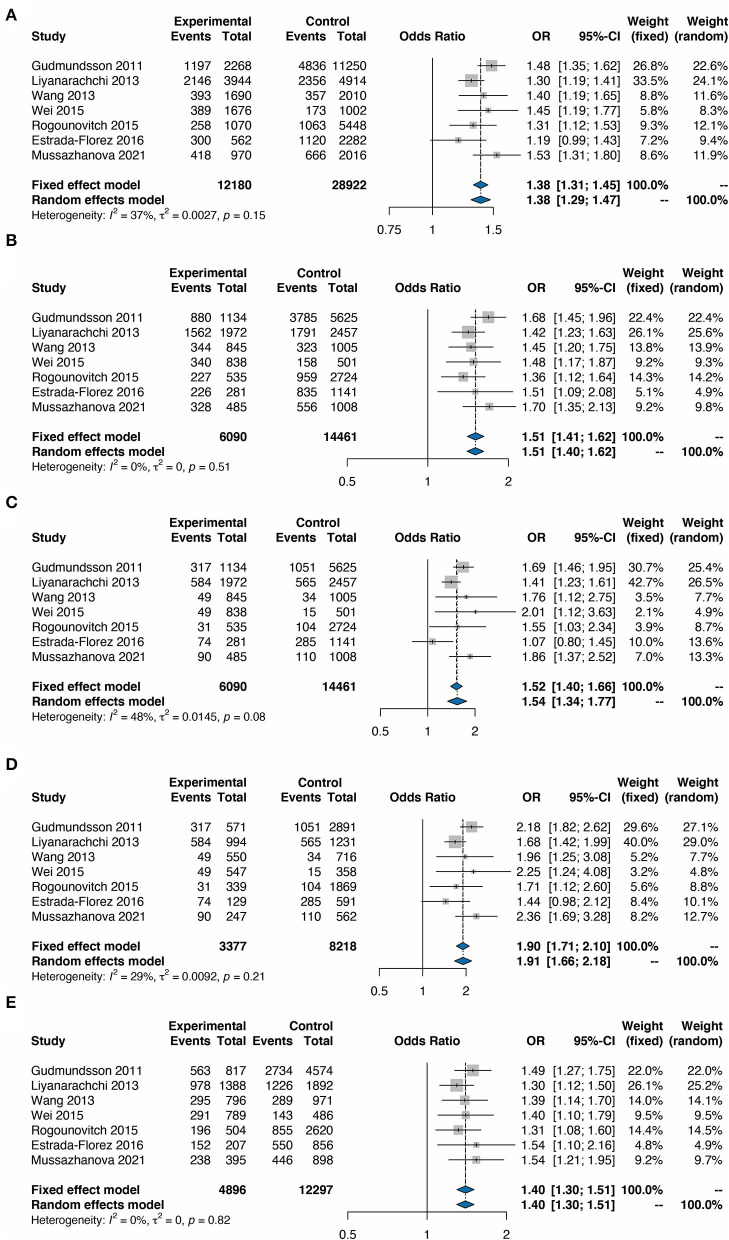
Forest plots for the meta-analysis of rs2439302 polymorphism and the risk of TC. **(A)** G vs. C (allele model). **(B)** GG plus CG vs. CC (dominant model). **(C)** GG vs. CC plus CG (recessive model). **(D)** GG vs. CC (homozygous model). **(E)** GC vs. CC (heterozygous model). OR, odds ratios; CI, confidence interval.

The fixed effect model was used to assess the overall ORs in all populations as well as in different countries, based on heterogeneity analysis. The heterogeneity analysis showed no significant heterogeneity in all the models including Allele, Dominant, Recessive, Homozygote, and Heterozygote models (*P* > 0.05). The TC risk correlated with the G allele was 1.38 times higher than that associated with the C allele (Figure 2A, OR = 1.38, 95% CI 1.31–1.45). In addition, the analysis based on the Dominant mode also indicated the significance of the correlation between rs2439302 and TC (Figure 2B, GG+GC/CC, OR = 1.51, 95% CI 1.41–1.62), Recessive model (Figure 2C, GG/CG+CC, OR = 1.52, 95% CI 1.40–1.66), Homozygote model (Figure 2D, GG/CC, OR = 1.90, 95% CI 1.71–2.10), and Heterozygote model (Figure 2E, GC/CC, OR = 1.40, 95% CI 1.30–1.51).

### Subgroup Analysis and Sensitivity Analysis

**Figure 3:**
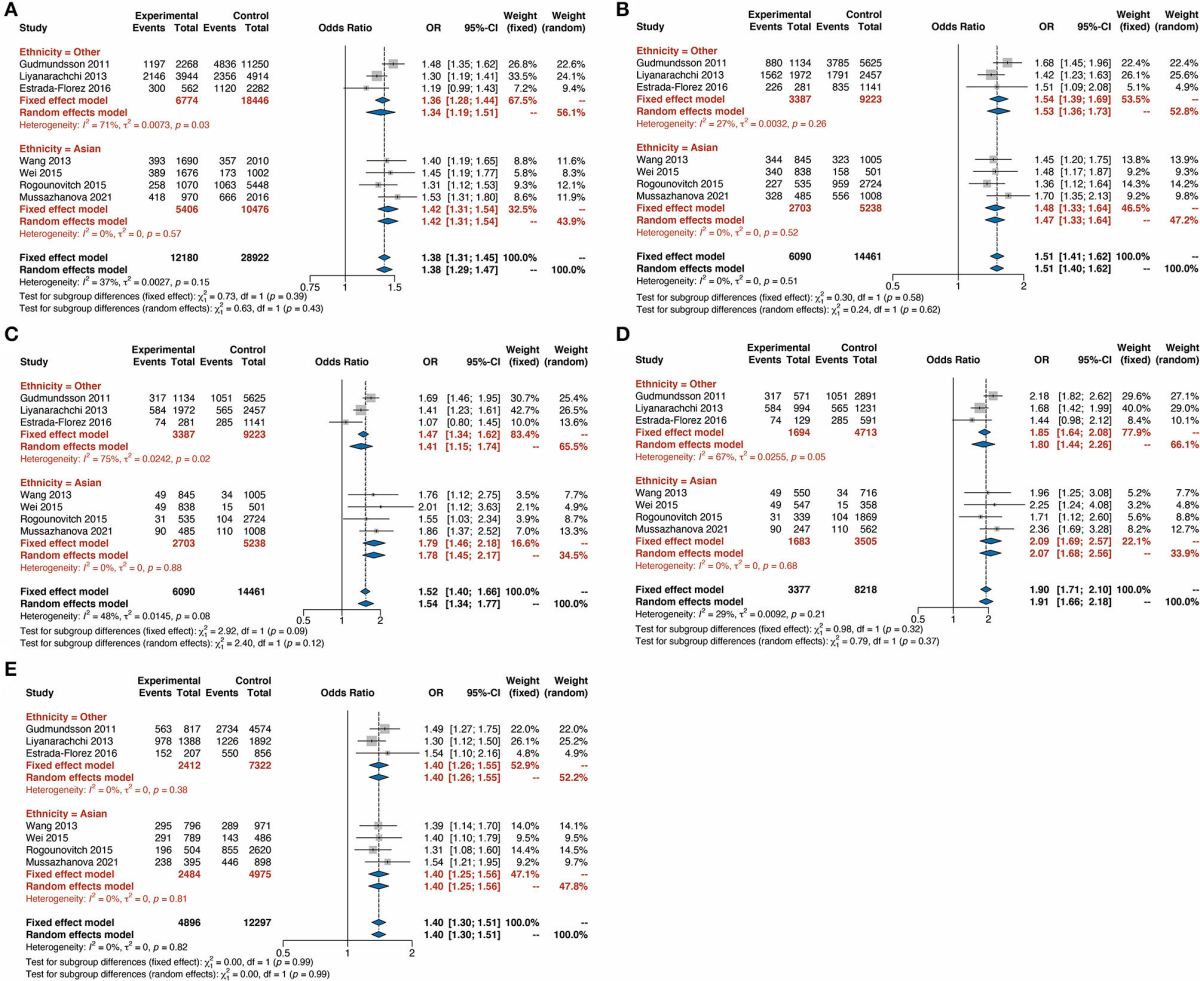
Forest plots for the subgroup-analysis of rs9929218 polymorphism and the risk of thyroid cancer based on ethnicity. **(A)** G vs. C (allele model). **(B)** GG plus CG vs. CC (dominant model). **(C)** GG vs. CC plus CG (recessive model). **(D)** GG vs. CC (homozygous model). **(E)** GC vs. CC (heterozygous model). OR, odds ratios; CI, confidence interval.

To further validate our findings, we conducted a subgroup analysis based on ethnicity. It was shown that the TC risk was significantly associated with the G allele compared with the C allele with an OR of 1.34 (95% CI 1.19–1.51) in other ethnicities and an OR of 1.42 (95% CI 1.31–1.54) in Asians (Figure 3A). The remarkable correlation between rs2439302 polymorphism and TC was also identified in the subgroup analysis in the Dominant model (Figure 3B, OR = 1.54, 95% CI 1.39–1.69 in other ethnicities, and OR = 1.48, 95% CI 1.33–1.64 in Asians), Recessive model (Figure 3C, OR = 1.41, 95% CI 1.15–1.74 in other ethnicities, and OR = 1.79, 95% CI 1.46–2.18 in Asians), Homozygote model (Figure 3D, OR = 1.85, 95% CI 1.64–2.08 in other ethnicities, and OR = 2.09, 95% CI 1.69–2.57 in Asians), and Heterozygote model (Figure 3E, OR = 1.40, 95% CI 1.26–1.55 in other ethnicities, and OR = 1.40, 95% CI 1.25–1.56 in Asians). These findings from the sub-group analysis revealed that Asiana and other populations with rs2439302 polymorphism showed a comparable high risk for TC.

We also conducted sensitivity analysis by omitting one of the included studies. The results indicated the significant association between rs2439302 polymorphism and TC, which was existed in all the five models in the sensitivity analysis (Supplementary Figure S1, OR > 1).

### Publication Bias Analysis and Sensitivity

**Figure 4:**
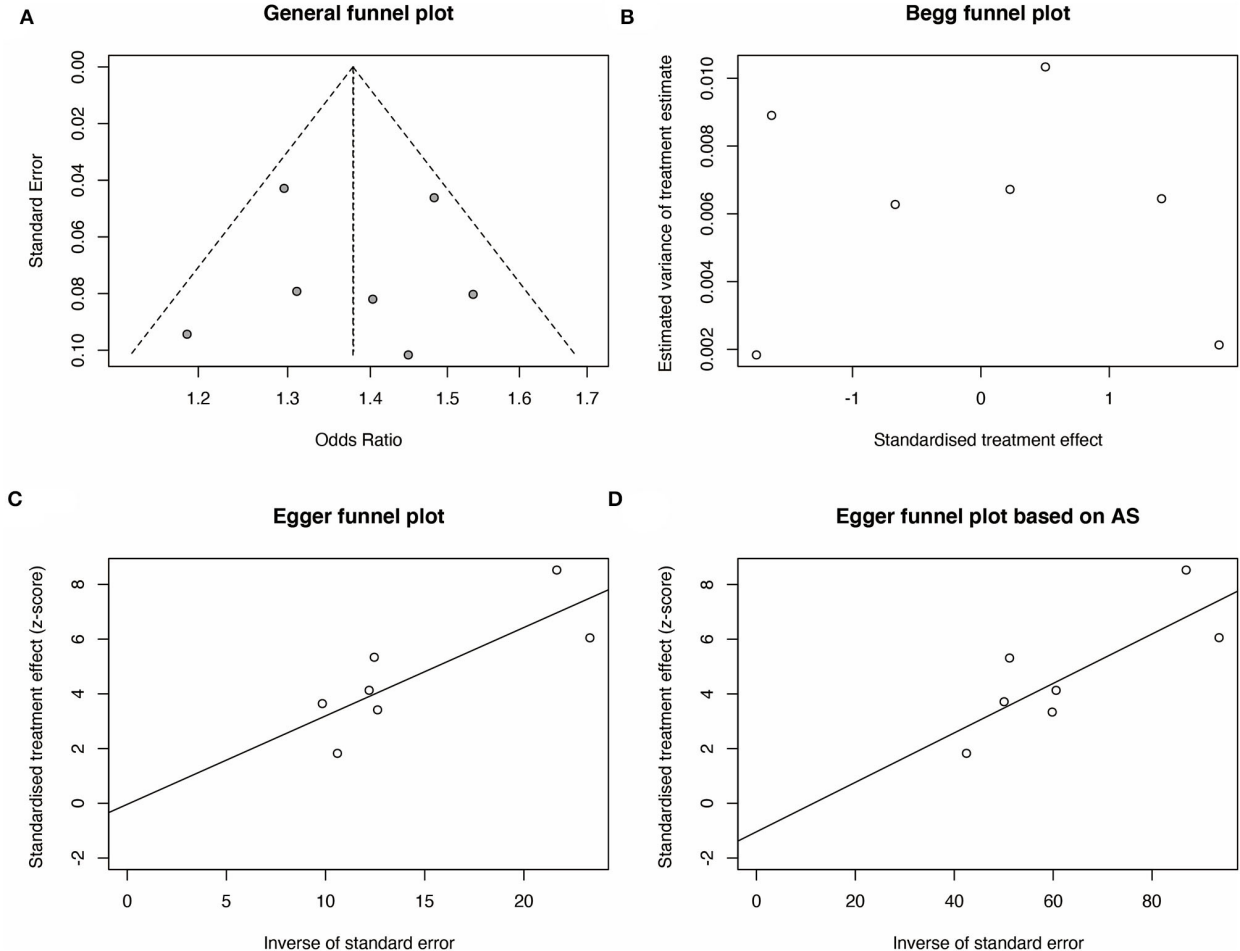
Publication bias analysis for rs2439302 polymorphism and the TC risk for G vs. C (allele model). **(A)** A funnel plot of publication bias analysis was performed to explore the correlation between rs2439302 polymorphism and TC risk. The methods based on linear regression proposed by **(B)** Begg plot and **(C)** Egger test were used to evaluate the asymmetry of the funnel plot. **(D)** The method based on linear regression proposed by the Egger test based on arcsine difference was utilized to analyze the asymmetry of the funnel plot.

We then carried out Begg's funnel plot and Egger's test to assess the publication bias of the studies. The funnel plots of the Allele, Dominant, Recessive, Heterozygote, and Homozygous models are symmetrical inverted funnels (Figure 4A, Supplementary Figures S2–S5), which suggest no significant publication bias. The results of both Begg's test and Egger's test were not significant (Figures 4B–D, *p* > 0.05). These findings revealed the stability and credibility of our conclusions of the meta-analysis.

## Discussion

Thyroid cancer is a multifactorial disease that involves genetic mutation and environmental changes (24). Currently, the 8p12 SNP rs2439302 has shown the strongest evidence of association with TC (11, 14–22). However, we noticed that no meta-analysis has been reported to analyze. The present study is the first comprehensive assessment of the literature focused on the correlation between rs2439302 polymorphism and TC.

Rs2439302 is located in the first intron of NRG1, a ligand for the ERBB protooncogene. It encodes a signal membrane protein which effects as a key regulator in the progression of various systems such as the nervous system, circulation system, and so on (25, 26). Additionally, NRG1 polymorphisms have been shown to be associated with schizophrenia, Alzheimer's disease, Hirschsprung's disease, TC, and other carcinoma development and metastasis (27–29). rs2439302 has been reported to influence NRG1 gene expression in the GTEx data, and Huiling et al. reported that the risk allele [G] is associated with the upregulation of NRG1; further, a DNA silencing of 32 kb containing the risk [G] allele of rs2439302 was revealed to harbor multiple candidate functional variants (21). However, Rogounovitch et al. determined the correlation between allele [G] of rs2439302 and the downregulation of NRG1; this could be because rs2439302 is located in the CTCF (CCCTC-binding factor, a transcription factor, and a highly conserved zinc-finger factor and DNA binding protein) binding region, and CTCF expression is decreased in TC tissues, which may result in the downregulation of NRG1 (14, 17). Nevertheless, the common ground for these studies is that rs2439302 has a role in the predisposition to TC. Jendrzejewski et al. showed that rs2439302 is correlated with lymph node metastasis (OR = 1.24, *p* = 0.016), and multifocality status of the tumor (OR = 1.24, *p* = 0.012) (20); Further, Estrada-Florez et al. indicated a higher association between rs2439302 and large tumors (OR = 1.50 *P* = 0.038) (11). The abovementioned study findings demonstrate that rs2439302 may be used effectively to identify patients with TC who are at the greatest risk.

In order to determine the TC risk under different genotypes, this meta-analysis analyzed the TC risk with rs2439302 based on different genetic models such as Allele (G/C), Heterozygote (GC/CC), Homozygote (GG/CC), Dominant (GG+GC/CC), and Recessive (GG/CG+CC). Results showed that the risk of TC associated with the G allele was 1.38 times higher than that of the C allele (OR = 1.38, 95% CI 1.31–1.45). In addition, this significant correlation between rs2439302 and TC also exists in the Dominant model (GG+GC/CC, OR = 1.51, 95% CI 1.41–1.62), Recessive model (GG/CG+CC, OR = 1.52, 95% CI 1.40–1.66), Homozygote model (GG/CC, OR = 1.90, 95% CI 1.71–2.10), and Heterozygote model (GC/CC, OR = 1.40, 95% CI 1.30–1.51). Subgroup analysis in different ethnicities was then carried out to investigate rs2439302 polymorphism in TC. The TC risk was significantly associated with the G allele compared with the C allele with an OR of 1.34 (95% CI 1.19–1.51) in other ethnicities and OR of 1.42 (95% CI 1.31–1.54) in Asians. It was also found that the rs2439302 polymorphism and TC were also significantly correlated based on subgroup analysis in the Dominant model, Recessive model, Homozygote model, and Heterozygote model. These findings from the sub-group analysis revealed that Asiana and other populations with rs2439302 polymorphism showed a comparable high risk for TC. Finally, the publication bias and sensitivity analysis indicated the stability of this meta-analysis.

Here, we found the vital association between rs2439302 and TC risk. However, some limitations still exist. First, owing to the lack of detailed information, the number of studies involved in this subject is small, which may lead to a lack of statistical capacity and hinder meaningful analysis of the results. Second, the effect of heterogeneity on the results could not be avoided even if a random-effects model was used. This heterogeneity may have been caused by factors such as a source of control, genotyping method, gene-environment interactions, and sample size. Third, although we found no publication bias *via* the Begg's and Egger's tests, the funnel plots of the Dominant and Recessive models were asymmetrical inverted funnels. Thus, publication bias may have been inevitable. Therefore, further analysis using larger sample size, a standardized unbiased method, and better-matched controls are required to obtain a more convincing conclusion.

Taken together, the present study indicated a significant association between rs2439302 and TC risk. Furthermore, we show that the Chinese populations have a higher risk than the Japanese and USA populations.

## Data Availability Statement

The original contributions presented in the study are included in the article/Supplementary Material, further inquiries can be directed to the corresponding author.

## Author Contributions

MG and JX conceived and designed the experiments. YG and WZ performed the search and collected the data. YG and RH analyzed the data. YG and CZ interpreted the results and drafted the manuscript. All authors approved the final manuscript as submitted and agree to be accountable for all aspects of the work.

## Funding

This work was supported by grants from the National Natural Science Foundation of China (81802674), Natural Science Foundation of Zhejiang Province (LY21H160049), Medical Health Science and Technology Project of Zhejiang Provincial Health Commission (2021KY482, 2020KY008), and Zhejiang Province Postdoctoral Research Excellence Funding Project (ZJ2021167). The funders had no role in study design, data collection and analysis, decision to publish, or preparation of the manuscript.

## Conflict of Interest

The authors declare that the research was conducted in the absence of any commercial or financial relationships that could be construed as a potential conflict of interest.

## Publisher's Note

All claims expressed in this article are solely those of the authors and do not necessarily represent those of their affiliated organizations, or those of the publisher, the editors and the reviewers. Any product that may be evaluated in this article, or claim that may be made by its manufacturer, is not guaranteed or endorsed by the publisher.
