# Supplementary material for: Investigating the Association Between rs2439302 Polymorphism and Thyroid Cancer: A Systematic Review and Meta-Analysis
Source: Front Surg. 2022 Apr 26;9:877206. doi: 10.3389/fsurg.2022.877206 (PMC9086625; doi:10.3389/fsurg.2022.877206)
Supplement: Supplementary file 1 [file Data_Sheet_1.pdf]

Supplementary Material

Supplementary Figures

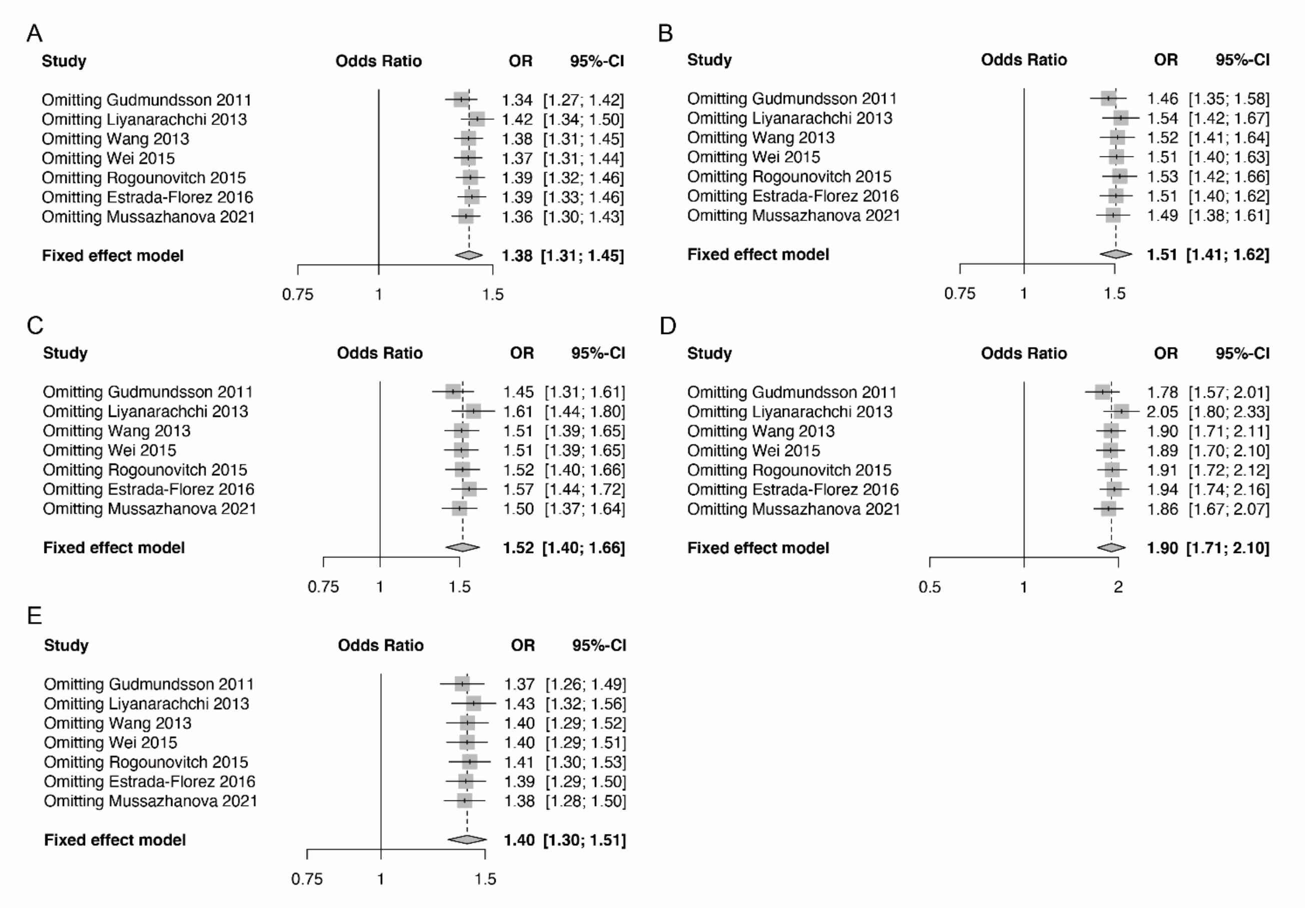

Supplementary Figure 1 Sensitivity analysis evaluating the correlation between rs9929218 polymorphism and the TC risk was carried out by omitting the selected publications. (A) G versus C (allele model). (B) GG plus CG vs CC (dominant model). (C) GG vs CC plus CG (recessive model). (D) GG vs CC (homozygous model). (E) GC vs CC (heterozygous model). OR, odds ratios; CI, confidence interval.

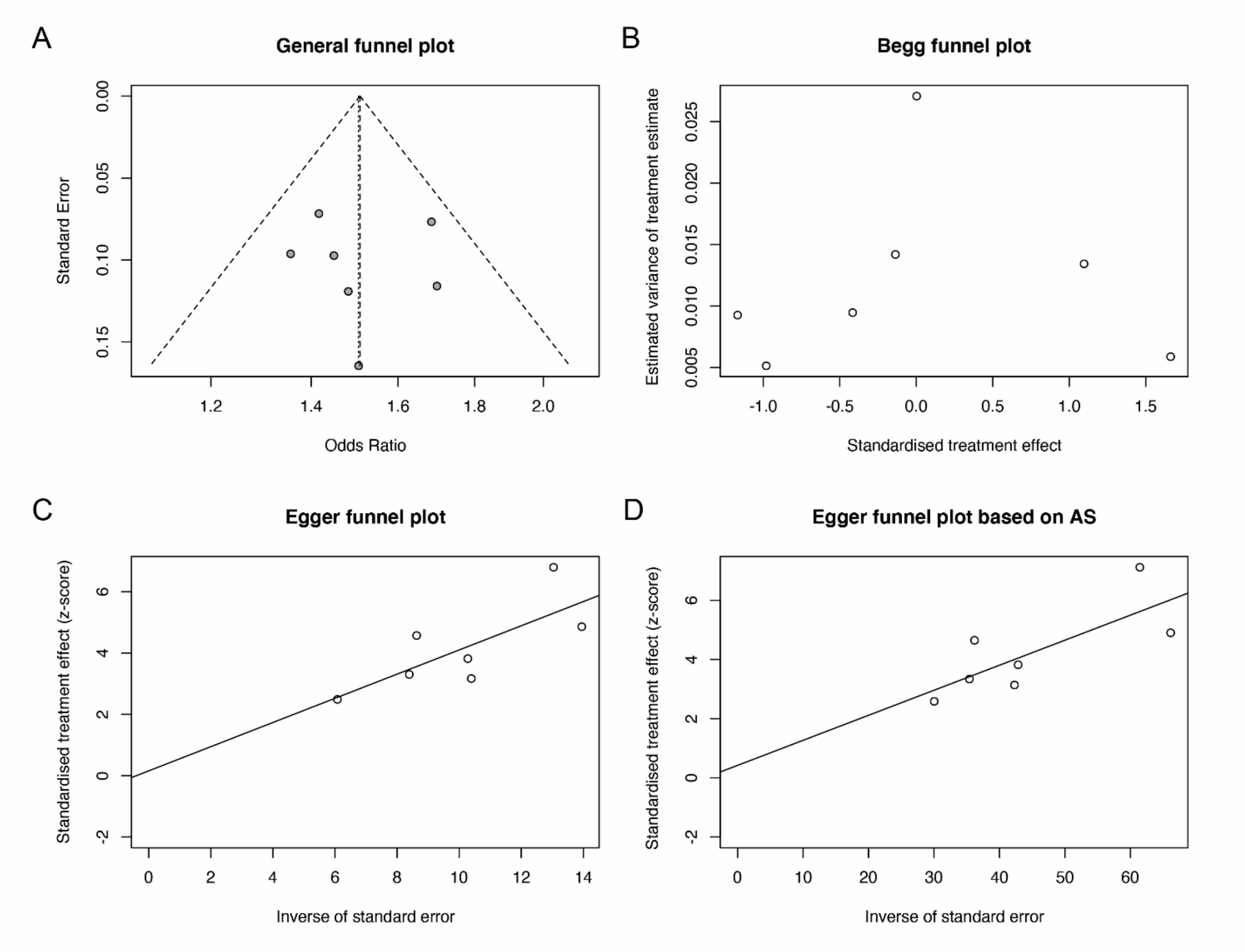

Supplementary Figure 2 Publication bias analysis for rs2439302 polymorphism and the TC risk for GG plus CG versus CC (dominant model). (A) Funnel plot for publication bias analysis of the selected studies was performed to explore the correlation between rs2439302 polymorphism and TC risk.The methods based on linear regression proposed by by (B) Begg plot and (C) Egger test were used to evaluate the asymmetry of the funnel plot. (D) The method based on linear regression proposed by Egger test based on arcsine difference is used to evaluate the asymmetry of the funnel plot.

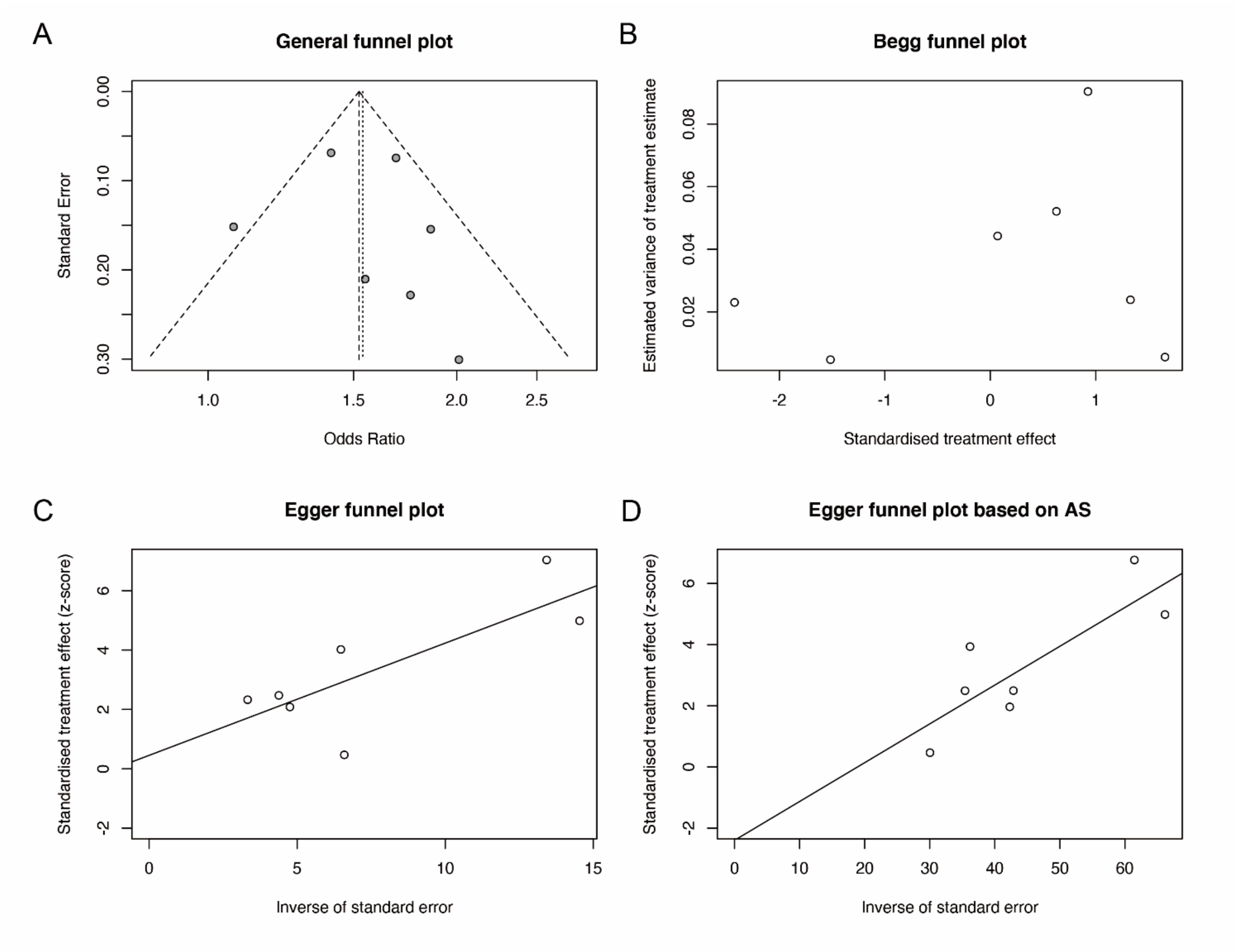

Supplementary Figure 3 Publication bias analysis for rs2439302 polymorphism and the TC risk for GG vs CC plus CG (recessive model). (A) Funnel plot for publication bias analysis of the selected studies was performed to explore the correlation between rs2439302 polymorphism and TC risk. The methods based on linear regression proposed by by (B) Begg plot and (C) Egger test were used to evaluate the asymmetry of the funnel plot. (D) The method based on linear regression proposed by Egger test based on arcsine difference is used to evaluate the asymmetry of the funnel plot.

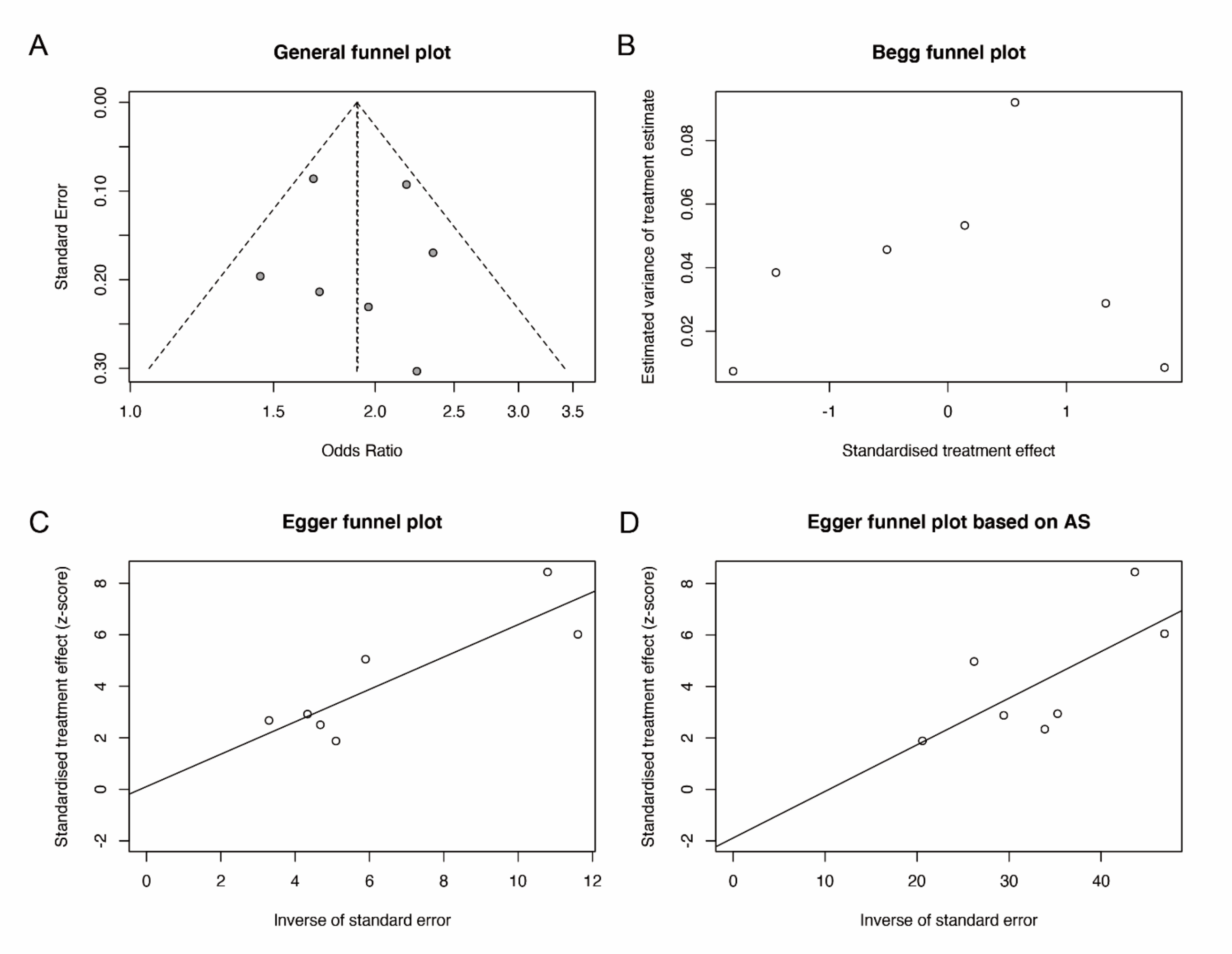

Supplementary Figure 4 Publication bias analysis for rs2439302 polymorphism and the risk of thyroid cancer for GG versus CC (homozygous model). (A) Funnel plot for publication bias analysis of the selected studies was performed to explore the correlation between rs2439302 polymorphism and TC risk. The methods based on linear regression proposed by by (B) Begg plot and (C) Egger test were used to evaluate the asymmetry of the funnel plot. (D) The method based on linear regression proposed by Egger test based on arcsine difference is used to evaluate the asymmetry of the funnel plot.

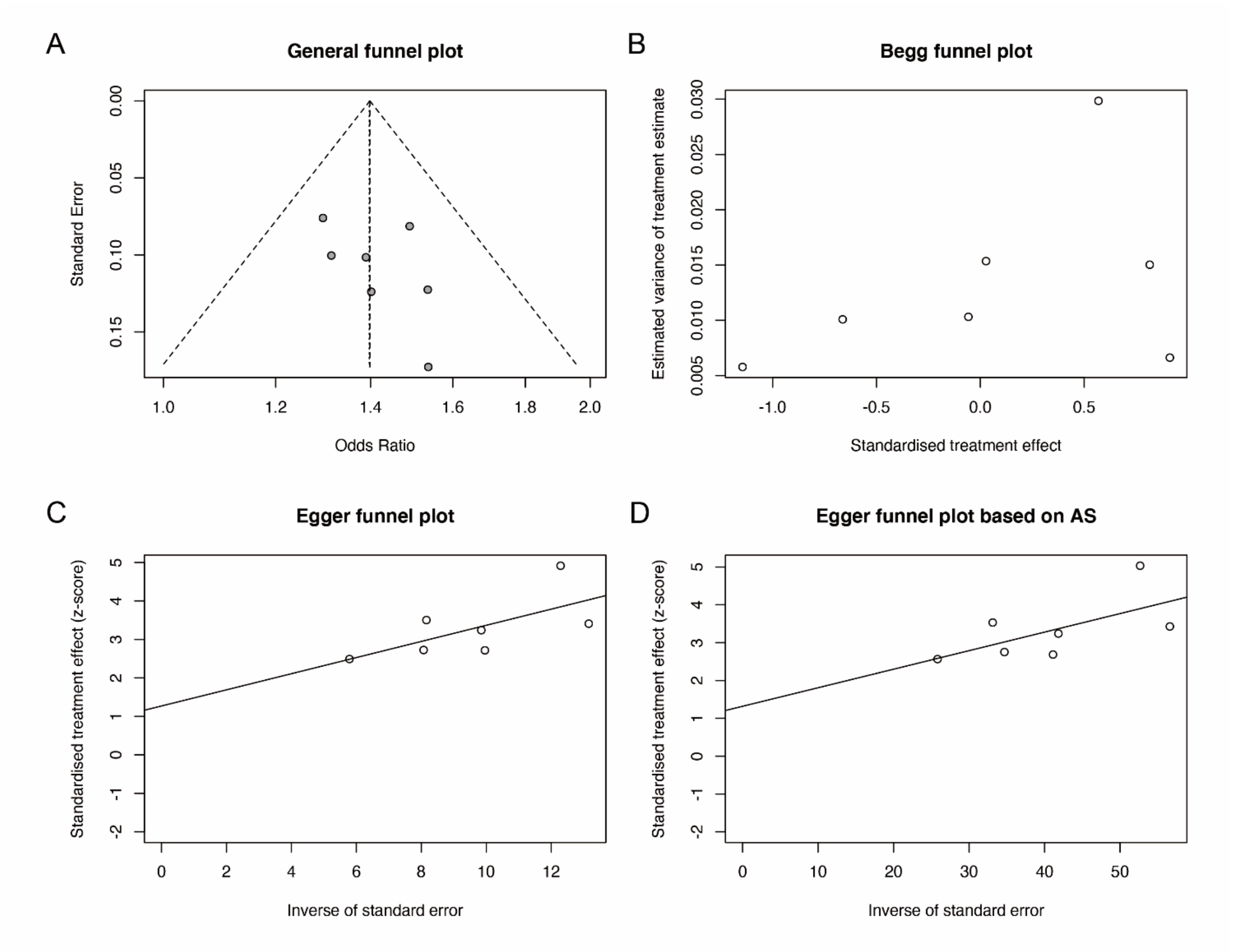

Supplementary Figure 5 Publication bias analysis for rs2439302 polymorphism and the risk of thyroid cancer for GC versus CC (heterozygous model). (A) Funnel plot for publication bias analysis of the selected studies was performed to explore the correlation between rs2439302 polymorphism and TC risk. The methods based on linear regression proposed by by (B) Begg plot and (C) Egger test were used to evaluate the asymmetry of the funnel plot. (D) The method based on linear regression proposed by Egger test based on arcsine difference is used to evaluate the asymmetry of the funnel plot.
